# Wnt1 oversees microglial activation by the Wnt/LRP5/6 receptor signaling pathway during lipopolysaccharide-mediated toxicity

**DOI:** 10.1007/s11033-025-10360-2

**Published:** 2025-03-01

**Authors:** Wang Qing, Xu Hao, Sun Xuan, Rong Zhihui, Gao Jinzhi

**Affiliations:** Department of Pediatrics, Tongji Hospital, Tongji Medical College, Huazhong University of Science and Technology, 1095 Jiefang Avenue, Wuhan, 430030 China

**Keywords:** β-Catenin, Autophagy, Wnt signaling, Microglia, Inflammatory

## Abstract

**Background:**

The protective effects of autophagy-mediated microglial inflammatory regulation on diseases of the central nervous system (CNS) has been a recent field of interest. The canonical signaling pathway activated by Wnt1, the Wnt/β-catenin signaling cascade, also plays a crucial protective role in neurodegenerative diseases. However, the relationship between Wnt1/β-catenin signaling and microglial activation remains unclear. Our study focused on understanding the impact and mechanism of Wnt1 on microglial activation.

**Methods and results:**

To simulate neuroinflammatory conditions in vitro, BV2 cells were exposed to 1 μg/mL lipopolysaccharide. CD86- and CD206-positive cells were identified by flow cytometry and immunofluorescence assays. Inflammatory and anti-inflammatory factors were measured using enzyme-linked immunosorbent assays. Autophagy was analyzed by expression of LC3B puncta, LC3, P62, and beclin1 expression. The inflammatory activation suppressed by rhWnt1 was restricted by DKK1, siRNA-β-catenin and siRNA-LKB1, respectively, with concomitant changes in β-catenin expression and phosphorylation of NFκB-p65, LKB1, and AMPK. Although the anti-inflammatory effect of Wnt1/LKB1 pathway was independent of β-catenin, Wnt1/LKB1 regulated β-catenin. The reduced inflammation caused by rhWnt1 is linked to its enhancement of autophagy, a process blocked by siRNA-LKB1 and 3-MA partially.

**Conclusions:**

The anti-inflammatory effects of Wnt1 on BV2 cells improved autophagy, a mechanism partly dependent on the β-catenin pathway or the phosphorylation of LKB1. Furthermore, the Wnt1/LKB1 pathway was activated independently of β-catenin and participated in regulating its expression. Our research unveils a previously unknown method through which Wnt1 exerts its anti-inflammatory effects, which may have a potential protective role against CNS diseases.

**Supplementary Information:**

The online version contains supplementary material available at 10.1007/s11033-025-10360-2.

## Introduction

In the central nervous system (CNS), microglia, which serve as the primary immune defense mechanism of the brain, may be activated by environmental signals and induce a pro-inflammatory or anti-inflammatory response. When pathogenic molecules promote the pro-inflammatory microglial response, the microglia activate into an M1-like phenotype, which then releases pro-inflammatory factors, leading to inflammatory damage of the CNS [1]. Conversely, protective molecules activate microglia into an M2-like phenotype, which contributes to anti-inflammatory cytokine production, enhance the growth and development of oligodendrocytes in laboratory settings, and start the process of remyelination within living organisms [2, 3]. Encouraging microglia to adopt an anti-inflammatory M2-like phenotype instead of a pro-inflammatory M1-like phenotype may reduce damage to nerve cells.

Wnt1, a cysteine-rich glycosylated protein related to the Drosophila protein Wingless, has been shown to inhibit microglial inflammatory activation in a variety of CNS diseases, such as ischemic reperfusion injury in the brain, intracerebral hemorrhage, Alzheimer’s disease, and Parkinson’s disease [4–7]. However, few studies have examined the specific mechanism of Wnt1 inhibiting microglial inflammatory activation. The Wnt/β-catenin signaling cascade represents a canonical example of the Wnt1 signaling pathway, which is also critical in regulating microglial phenotypes [8]. Our previous research has shown that Wnt1 overexpression induces an M2-like phenotype through the LKB1 signaling pathway [9]. The relationship between the Wnt/β-catenin signaling cascade and the LKB1 signaling pathway in microglia warrants further research.

Autophagy significantly influences the inflammatory activation of microglia [10–12], which also plays a critical role in the development of neurodegenerative diseases and ischemic stroke. Enhancing autophagy may alleviate CNS damage by regulating microglia activation [13]. Several studies have assessed how the Wnt/β-catenin signaling pathway influences autophagy regulation [14, 15]. Interactions between the Wnt/β-catenin pathway and autophagy mechanisms have been described [15]. However, whether the role of Wnt1 in microglial activation is associated with LKB1-mediated autophagy dependent on β-catenin signaling or not is not fully understood.

In postmortem analyses of white matter injury (WMI) in premature infants, microglia accumulate in diffuse regions of the brain, which were found to be M1-like [2]. Wnt1 inhibiting microglial inflammatory activation, might participate in the protection of myelinogenesis and neurons in WMI. Gram-negative bacteria are the main causes of bacterial infections during pregnancy. Lipopolysaccharide (LPS), an important component of the cell wall of Gram-negative bacteria, has been shown to frequently cause fetal sepsis and is widely used to induce systemic inflammation and WMI in newborn rat models [16]. Here, we promoted BV2 cells to inflammatory activation using low-concentration LPS, as described in previous studies [17], to mimic the inflammatory state of WMI in preterm infants in vitro. We used DKK1 to inhibit the binding of Wnt1 and LRP5/6 receptors to elucidate the role of the canonical pathway in Wnt1 regulating microglial activation. Targeted silencing of β-catenin and LKB1 was conducted to investigate whether their downstream signaling pathways are interdependent. Changes related to the Wnt/β-catenin and LKB1 signaling pathways and autophagy at the molecular level were identified. Our research focused on exploring how Wnt1 influences microglial inflammation and clarifying the subsequent signaling pathways involved.

## Methods

### Microglial cell culture

The BV2 mouse microglial cell line, a well-established model, was obtained from the China Center for Type Culture Collection located in Wuhan, China. BV2 cells were cultured using Dulbecco’s modified Eagle medium (DMEM, Hyclone, Cytiva), supplemented with 10% fetal bovine serum (Gibco, Thermo Fisher Scientific). The cells were plated at a concentration of 1 × 10^5^ cells per mL and maintained in an environment of 37 °C, 5% CO_2_, and 95% humidity. Approximately 4 h prior to the experiment, the medium was refreshed with new DMEM.

To determine the optimal concentration for future experiments, BV2 cells underwent a 2-h incubation period with increasing doses of LPS (0, 0.5, 1, 5, 10 μg/mL; Sigma-Aldrich). To investigate how Wnt1 influences inflammatory responses in BV2 cells, including the underlying mechanisms involved, recombinant human Wnt1 protein (rhWnt1, 50 ng/mL; Fitzgerald Industries International, Gardner, MA), Dickkopf-related protein 1 (DKK1, LRP5/6 receptor antagonist, 100 ng/mL; Beyotime Biotechnology, China), and 3-methyladenine (3-MA, 500 μM; Sigma-Aldrich) were used to treat cells approximately 2 h before LPS treatment in subsequent experiments.

### Cell viability assay

The Cell Counting Kit-8 (CCK-8) from Beyotime Biotechnology is frequently utilized for straightforward and precise assessments of cell growth and toxicity. BV2 cells were grown on 96-well plates for 24 h. Following this period, they were treated with various concentrations of LPS for an additional 2 h. Afterwards, the CCK-8 reagent was added to each well and the culture was incubated for 2 h. Medium infused with solvent was used to fill respective blanks. A microplate reader then measured the absorption at 450 nm (Diatek, China). An Olympus microscope was used to image cells at ×20 magnification (Tokyo, Japan).

### Silencing β-catenin and LKB1 gene expression by using siRNA

We obtained siRNA agents targeting the β-catenin and LKB1 genes from Cell Signaling Technology (Danvers, MA) to suppress the expression of these genes. Scrambled siRNA (siRNA NC) duplexes were used as the control. When BV2 cells reached 60–80% confluence, the siRNA duplexes were transfected into cells using Lipofectamine 2000 (Invitrogen, Carlsbad, CA). Seventy-two hours after transfection, the cells were collected for additional experiments.

### ELISA for cytokine analysis

Cytokines (IL-1β, IL-6, IL-4, IL-10) secreted into the culture supernatant were collected and measured. Enzyme-linked immunosorbent assays (ELISAs) were carried out following the kit instructions (ELK Biotechnology, China). A microplate reader was used to determine the optical density at 450 nm, whereas cytokine concentrations were quantified based on a standard curve.

### Flow cytometry

Cells were incubated, as previously mentioned, in 6-well plates and then digested using trypsin (Sigma-Aldrich). The isolated cells were washed and resuspended using cold phosphate-buffered saline (PBS) at a concentration of 0.01 mol/L. We mixed a 100 μL cell suspension, containing 1 × 10^7^ cells/mL, with APC anti-mouse CD86 antibody (Thermo Fisher Scientific) at a ratio of 1:50. This mixture was then incubated at 4 °C in the dark for 30 min. Subsequently, the cells were rinsed using PBS, fixed in 200 μL of solution, and incubated again at 37 °C for 20 min away from light. After removing the impurities, the cells were re-suspended in 1 mL of a lysing solution and then kept in the dark at 4 °C for 30 min to ensure proper incubation. Following centrifugation, 100 μL of a lysing solution was used to resuspend the cells, which were then exposed to PE anti-mouse CD206 antibody (1:50, BioLegend, San Diego, CA) and incubated at 4 °C in the dark for 30 min. Following the initial preparation, the cells were rinsed with PBS. They were then resuspended in 100 μL of PBS. For the analysis, a BeckmanCytoFLEX flow cytometer (Beckman Coulter, Brea, CA) was employed.

### Immunofluorescence staining

Approximately 24 h prior to the culture and treatment of BV2 cells, slides were coated with poly l-lysine (0.1 mg/mL, Sigma-Aldrich). The cells were fixed using 4% paraformaldehyde, were then permeabilized with 0.3% Triton X-100, and subsequently blocked with 3% donkey serum. Cells were incubated overnight at 4 °C with primary anti-mouse CD86 antibodies (1:100, Novus, Centennial, CO) and anti-rabbit CD206 antibodies (1:100, Abcam, Cambridge, UK). The following day, the cells were treated with secondary goat anti-mouse CoraLite488® antibodies (Proteintech, Rosemont, IL, 1:100) and goat anti-rabbit CY3® antibodies (Aspen, China, 1:100) at a temperature of 4 °C for 1 h. To visualize the nuclei, 4′,6-Diamidino-2′-phenylindole staining was applied. Cells stained and then sealed with anti-fade mounting medium were examined using an Olympus fluorescence microscope, model BX51 AX-70. Similar to the procedures described above, the primary anti-rabbit LC3B (1:500, Proteintech) and secondary goat anti-rabbit CY3® (1:100. Aspen) antibodies were incubated with treated BV2 cells. A confocal microscope (LSM700; Carl Zeiss, Jena, Germany) was used to count the number of LC3B puncta in each cell.

### Western blotting

The protein lysate was prepared using cell lysis buffer (Beyotime Biotechnology) and phenylmethylsulfonyl fluoride (Sigma-Aldrich) according to the manufacturer’s instructions. A nuclear cytosol extraction kit (Applygen, China) was used to prepare nuclear extracts, and the extracted protein was quantified with a BCA protein assay kit (Beyotime Biotechnology). Proteins, each loaded at 40 μg per lane, and were separated using 10% sodium dodecyl sulfate polyacrylamide gel electrophoresis before being transferred onto polyvinylidene fluoride membranes. The membranes were first treated with 5% skim milk as a blocking agent. They were then left to incubate at a temperature of 4 °C throughout the night, using a different rabbit primary polyclonal antibodies. The antibodies used included β-catenin at a dilution of 1:3000 (Abcam), phospho-NFκB-p65, NFκB-p65, phospho-AMPKα, and AMPKα (all from Cell Signaling Technology) each at dilutions of 1:500 and 1:2000, respectively phospho-LKB1, and LKB1 (Proteintech) at 1:500 and 1:3000. Additionally, Beclin1, P62, LC3, and GAPDH were also used at dilutions of 1:2000, 1:2000, 1:1000, and 1:10,000, respectively; all antibodies were sourced from Cell Signaling Technology and Abcam. Samples were subsequently treated with horseradish peroxidase-conjugated goat anti-rabbit secondary antibodies (Aspen, 1:10,000) and maintained at room temperature for 2 h. The protein bands were quantified with an enhanced chemiluminescence detection system and the results were processed through ImageJ software (National Institutes of Health, Bethesda, MD). Protein expression was adjusted based on GAPDH normalization, showing fold changes in comparison with the control group.

### Co-immunoprecipitation assay

Cells were incubated, as previously mentioned, in 6-well plates and then harvested using 400 μL lysis buffer (NP-40 RIPA: cocktail = 100:1, Beyotime Biotechnology). For each 500-µg protein sample, specific primary antibodies were utilized: anti-LKB1 at a 1:500 dilution from Proteintech, anti-MO25 at a 1:50 dilution from Cell Signaling Technology, and anti-rabbit IgG at a 1:200 dilution, also from Proteintech. These samples were rotated continuously overnight at a temperature of 4 °C. Following sample preparation, we incorporated 100 µL of protein A/G beads and continuously rotated the mixture at a temperature of 4 °C for an additional hour to ensure the capture of the conjugated polymers. The beads, once collected by centrifugation and treated with RIPA buffer, were then resuspended in 40-µL of RIPA buffer and denatured using a 5× loading buffer. The immunoprecipitated complexes were subjected to Western blot analysis, following the methods outlined earlier. The antibodies used were as follows: anti-LKB1 (Proteintech) at a dilution of 1:3000, anti-MO25 (Cell Signaling Technology) diluted 1:1000, and anti-STRAD (Abcam) at a dilution of 1:2000.

### Statistical analysis

Values were reported as the mean ± the standard deviation, based on data collected from at least three separate experiments. Statistical evaluations were conducted using SPSS v.17.0 (IBM, Armonk, NY). Statistical significance was determined using one-way analysis of variance and Student’s t-test, with differences considered significant at P values less than 0.05.

## Results

### Effects of LPS on the activation of BV2 cells

Microglia were activated to an inflammatory state when exposed to LPS. BV2 cells were exposed to increasing concentrations of LPS (0, 0.5, 1, 5, and 10 μg/mL) for 2 h. With increasing LPS concentration, microglia showed enlarged cell bodies, enlarged proximal antenna, and reduced branching of the distal antenna (Fig. S1A). A significant reduction in cell viability was observed using the CCK-8 assay when the LPS concentration reached 5 μg/mL (Fig. S1B). LPS at 5 µg/mL reduced the cell viability to 81.18% (P < 0.05); at 10 µg/mL, it reduced the cell viability to 50.43% (P < 0.05). Additionally, ELISAs identified the release of pro-inflammatory cytokines (IL-1β, IL-6). LPS at 0.5 μg/mL did not significantly increase the IL-1β concentration (P > 0.05). However, at concentrations above 0.5 μg/mL, the higher the concentration, the more IL-1β was secreted (P < 0.05) (Fig. S1C). As the LPS concentration rose, a corresponding gradual increase in the levels of IL-6 was observed (P < 0.05) (Fig. S1D). Given the observed cell viability and levels of inflammatory factors, 1 μg/mL of LPS was chosen for use in future experiments.

### Wnt1 restricted inflammatory activation of BV2 cells through the canonical pathway to some extent

**Fig. 1:**
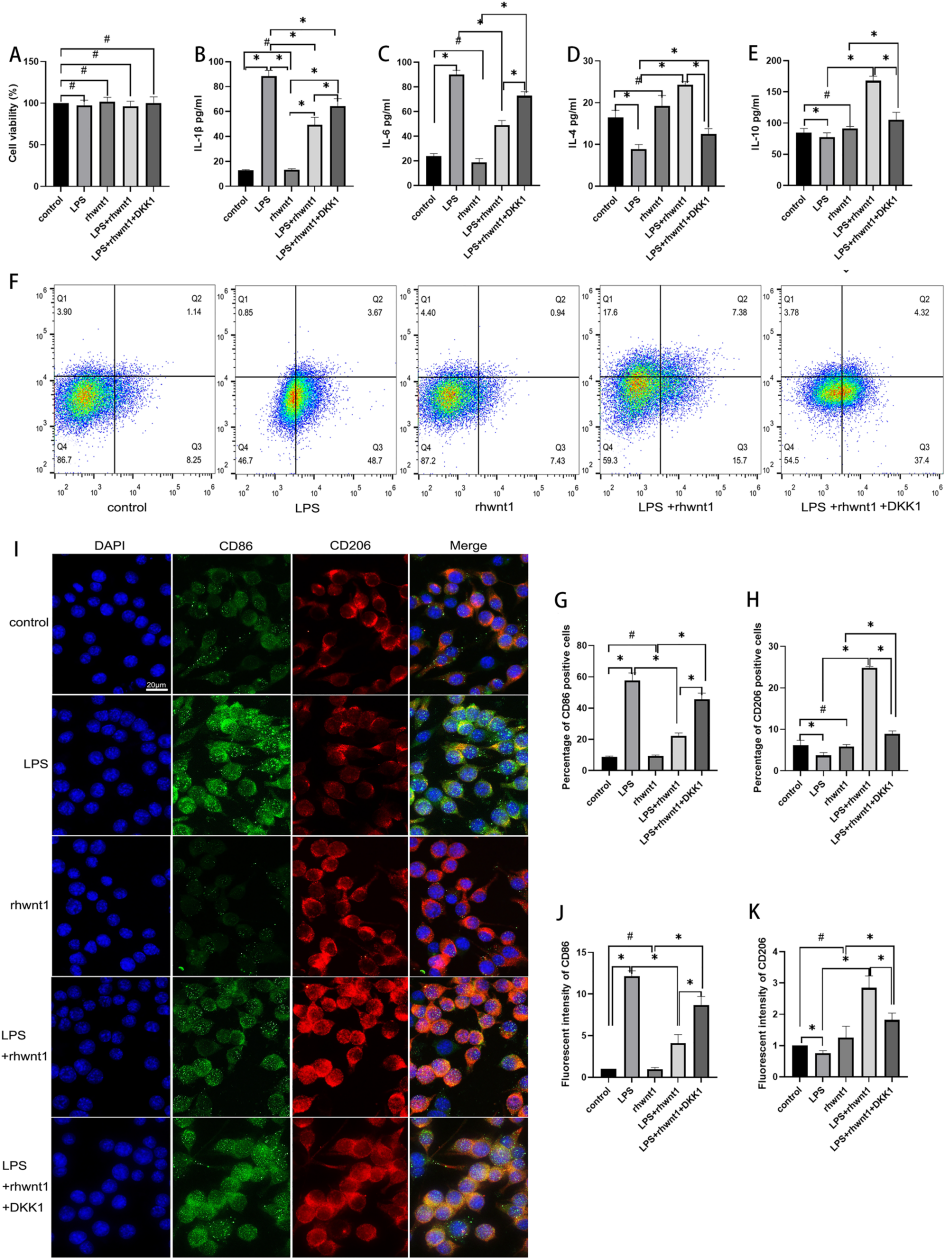
Wnt1 restricted the inflammatory activation of BV2 cells through the canonical pathway. Cell viability was detected by the CCK-8 assay. No difference in cell viability across all groups was observed (**A**). The secretion of cytokines (IL-1β, IL-6, IL-4, IL-10) was detected by ELISA. rhWnt1 alone did not affect cytokine secretion. rhWnt1 reduced the levels of IL-1β and IL-6 secreted by LPS-treated BV2 cells (**B**, **C**) and enhanced the release of IL-4 and IL-10 by LPS-treated BV2 cells (**D**, **E**). The above effects were partially reversed by the addition of DKK1 (**B**–**E**). The proportion of CD86-positive and CD206-positive cells were assayed by flow cytometry and immunofluorescence. rhWnt1 did not influence the percentages of CD86-positive and CD206-positive cells in the absence of LPS (**F**–**K**). rhWnt1 reduced the LPS-induced increase in percentage of CD86-positive cells and augmented the LPS-reduced percentage of CD206-positive cells (F-K). The reduction in CD86-positive cells and increase in CD206-positive cells caused by rhWnt1 were partially blocked by DKK1 (a LRP5/6 receptor antagonist) (**F**–**K**). *P < 0.05, #P > 0.05. green: CD86-positive cells, red: CD206-positive cells. LPS, lipopolysaccharide; ELISA, enzyme-linked immunosorbent assay

Neither LPS, rhWnt1 nor DKK1 altered BV2 cell viability (P > 0.05) (Fig. 1A). The levels of proinflammatory cytokines (IL-1β and IL-6) and anti-inflammation cytokines (IL-4 and IL-10) were not affected by rhWnt1 intervention (P > 0.05) (Fig. 1B–E). However, in LPS-treated BV2 cells, rhWnt1 reduced the concentrations of IL-1β and IL-6 from 88.46 ± 4.61 pg/mL and 90.05 ± 2.85 pg/mL to 49.40 ± 5.95 pg/mL and 49.16 ± 2.93 pg/mL (P < 0.05) (Fig. 1B, C), and simultaneously increased the release of IL-4 and IL-10 from 8.94 ± 0.86 pg/mL and 77.31 ± 5.86 pg/mL to 24.26 ± 0.69 pg/mL and 167.93 ± 5.90 pg/mL (P < 0.05) (Fig. 1D, E). Conversely, in LPS-treated BV2 cells, the rhWnt1-induced reduction of IL-1β and IL-6 concentrations or increased IL-4 and IL-10 concentrations were partially reversed by the addition of DKK1 (P < 0.05) (Fig. 1B–E).

CD86 is found predominantly in pro-inflammatory M1-like microglia, while CD206 is mainly associated with anti-inflammatory M2-like microglia. Flow cytometry showed that rhWnt1 did not influence the activation of BV2 cells in the absence of LPS (P > 0.05) (Fig. 1F–H). However, LPS-induced increase of CD86-positive cells was notably reduced by exposure to rhWnt1 (P < 0.05) (Fig. 1F–H). In contrast, when LPS reduced the number of CD206-positive cells, rhWnt1 significantly increased their proportion (P < 0.05) (Fig. 1F–H). The reduction in CD86-positive cells and the increase in CD206-positive cells caused by rhWnt1 were partially blocked by DKK1 (P < 0.05) (Fig. 1F–H).

Flow cytometry results were corroborated by immunofluorescence assays, demonstrating consistent changes in the ratio of CD86 to CD206 cells across all groups (Fig. 1I–K).

### Wnt1 regulated β-catenin expression and phosphorylation of NFκB, LKB1, and AMPK through the canonical pathway

**Fig. 2:**
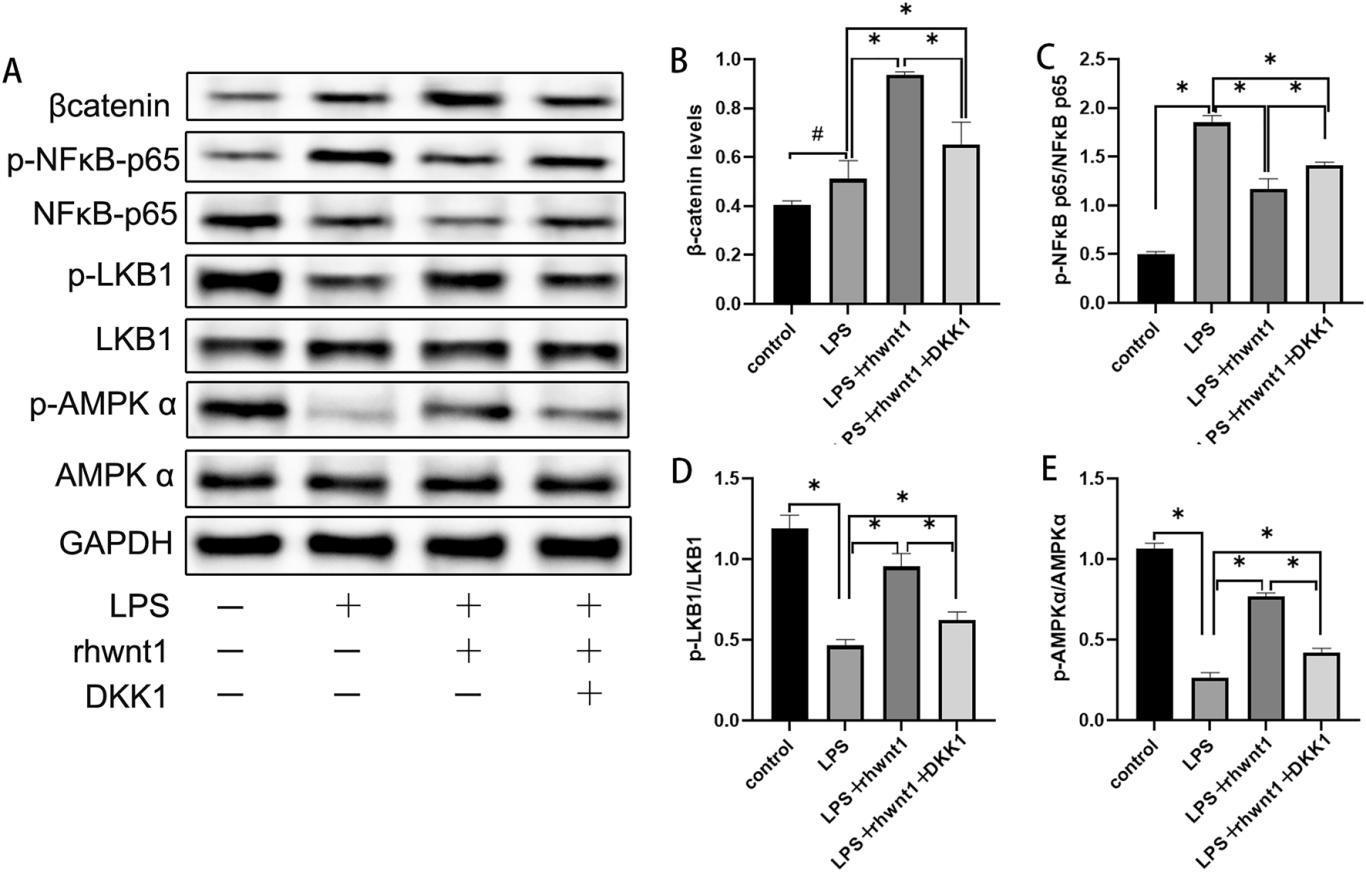
Wnt1 regulated β-catenin expression and phosphorylation of NFκB, LKB1, and AMPK in an LRP5/6 receptor-dependent manner. The LPS-induced increase of β-catenin expression was not significant when compared with the control group. LPS and rhWnt1 treatment significantly increased the expression level of β-catenin, which was decreased after blocking with DKK1 (**A**, **B**). LPS increased the level of NFκB-p65, which was restrained by rhWnt1, whereas changes in the levels of phosphorylation of LKB1 and AMPK were just opposite to those of NFκB-p65 (**A**, **C**–**E**). DKK1 blocked changes in signaling molecules downstream of rhWnt1 (**A**, **C**–**E**). *P < 0.05, #P > 0.05 LPS, lipopolysaccharide

Following LPS-induced activation of inflammation, NFκB-p65 phosphorylation was increased and LKB1 and AMPK phosphorylation was decreased (P < 0.05) (Fig. 2A, C–E). LPS did not significantly alter β-catenin levels (P > 0.05) (Fig. 2A, B). In LPS-treated BV2 cells, β-catenin expression was increased by rhWnt1 and this increase was abrogated by DKK1 (P < 0.05) (Fig. 2A, B). In LPS-treated BV2 cells, besides the restrained inflammatory activation by rhWnt1, upregulation of NFκB-p65 phosphorylation, downregulation of LKB1, and AMPK phosphorylation were all reversed accordingly (P < 0.05) (Fig. 2A, C–E). DKK1 not only inhibited the anti-inflammatory effects of rhWnt1 but also suppressed the phosphorylation of NFκB-p65, LKB1, and AMPK induced by rhWnt1 (P < 0.05) (Fig. 2A, C–E). LKB1 and AMPK could be downstream signaling molecules of Wnt1 depending on β-catenin.

### Screening of LKB1- and β-catenin-specific siRNA

Three distinct sequences targeting LKB1 and β-catenin are included in commercial siRNA reagents. The effectiveness of each siRNA sequence in silencing was confirmed by Western blot analysis. Among these, siRNA-β-catenin-1 demonstrated the highest silencing efficiency (P < 0.05) (Fig. S2A, E). After exposure to siRNA-β-catenin-1, the expression of β-catenin protein was reduced to just 10.86% compared with the control group (P < 0.05). siRNA-LKB1-2 was the most effective for silencing LKB1, with protein levels reduced to just 10.06% of those of the control group (P < 0.05) (Fig. S2B, F). The CCK-8 assay revealed no notable differences in cell viability across the various siRNA intervention and control groups (P > 0.05) (Fig. S2C, D).

### Targeted silencing of β-catenin or LKB1 expression partially suppressed the anti-inflammatory effects of rhWnt1

**Fig. 3:**
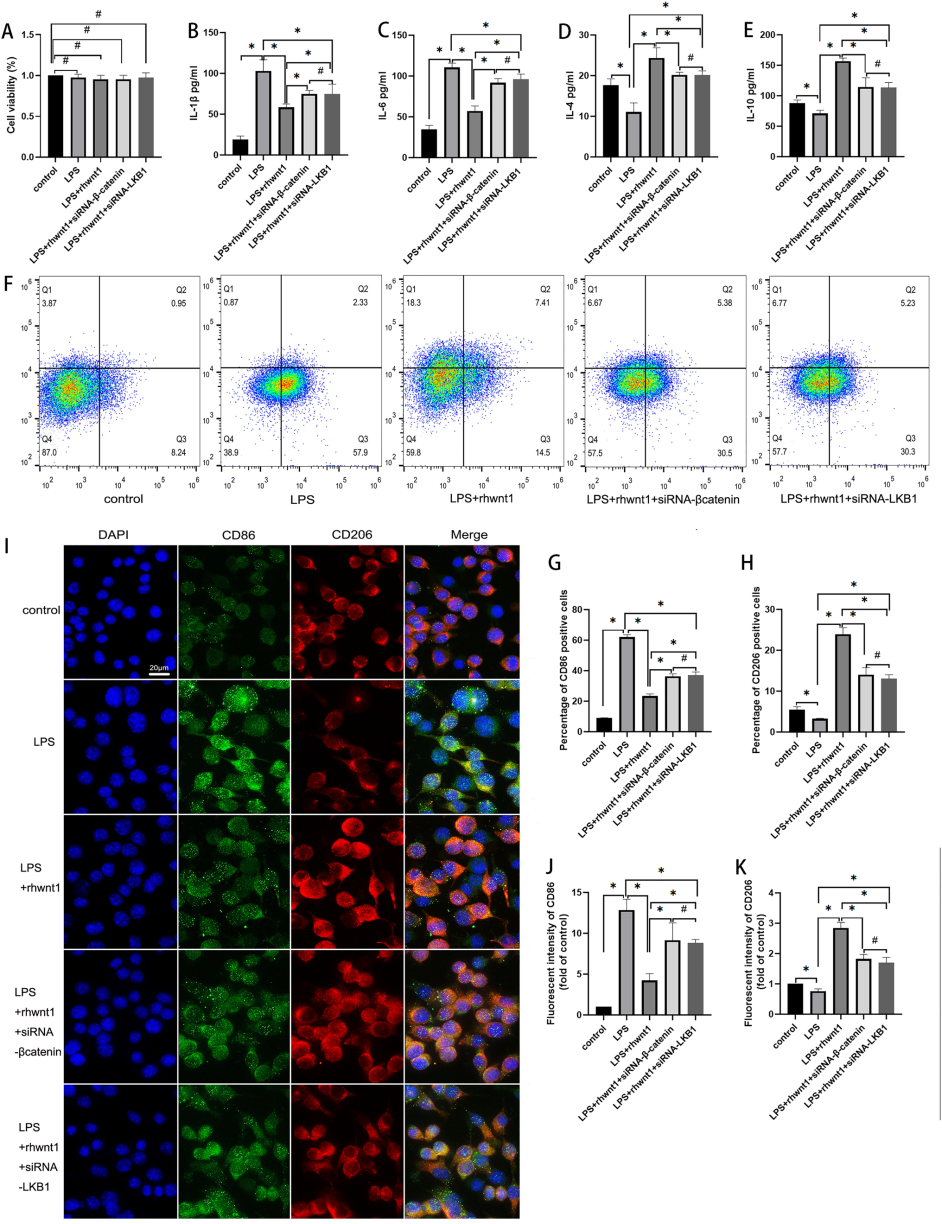
The anti-inflammatory effects of rhWnt1 on BV2 cells were partially suppressed by siRNA-β-catenin and siRNA-LKB1. The CCK8 assay showed that cell viability was not affected by all treatments (**A**). Pro- and anti-inflammatory factors were detected by ELISA. rhWnt1 reversed not only the LPS-induced increase of IL1-β and IL-6, but also the LPS-induced decrease of IL-4 and IL-10. These reversals were partially blocked by siRNA-β-catenin and siRNA-LKB1 (**B**–**E**). Flow cytometry showed that rhWnt1 decreased the proportion of LPS-induced CD86-positive cells and increased the LPS-induced downregulation of CD206-positive cells. Both siRNA-β-catenin and siRNA-LKB1 partially inhibited these effects (**F**–**H**). Immunofluorescence indicated that the changes in the fluorescent intensity of CD86 and CD206 in all groups were consistent with the flow cytometry findings (**I**–**K**). Furthermore, no significant difference was observed in the inflammatory activation of BV2 cells between the LPS + rhWnt1 + siRNA-β-catenin and LPS + rhWnt1 + siRNA-LKB1 groups. *P < 0.05, #P > 0.05 LPS, lipopolysaccharide; ELISA, enzyme-linked immunosorbent assay

Neither LPS, rhWnt1, siRNA-β-catenin, nor siRNA-LKB1 influenced the viability of BV2 cells (P > 0.05) (Fig. 3A). Moreover, siRNA-β-catenin and siRNA-LKB1 restricted the anti-inflammatory effects of rhWnt1. After LPS stimulation, the IL-1β concentration increased from 19.24 ± 4.05 pg/mL to 102.93 ± 13.87 pg/mL and IL-6 levels increased from 34.45 ± 5.16 pg/mL to 110.56 ± 5.25 pg/mL, whereas rhWnt1 reduced IL-1β and IL-6 concentrations to 58.39 ± 4.13 pg/mL and 57.06 ± 6.38 pg/mL, respectively (P < 0.05) (Fig. 3B, C). After LPS stimulation, IL-4 levels decreased from 17.69 ± 1.55 pg/mL to 11.07 ± 2.25 pg/mL and IL-10 levels decreased from 87.76 ± 5.28 pg/mL to 71.14 ± 5.09 pg/mL, whereas rhWnt1 reversed IL-4 and IL-10 concentrations to 24.37 ± 2.5 pg/mL and 156.64 ± 5.3 pg/mL, respectively (P < 0.05) (Fig. 3D, E). When BV2 cells were exposed to siRNA-β-catenin plus LPS and rhWnt1 simultaneously, the IL-1β and IL-6 concentrations were higher than those in the rhWnt1 plus LPS intervention group, but were still lower than those exposed to LPS alone (P < 0.05) (Fig. 3B, C); the IL-4 and IL-10 concentrations were lower than those in the rhWnt1 plus LPS intervention group but still higher than those in the LPS intervention group (P < 0.05) (Fig. 3D, E). Similar results were observed for siRNA-LKB1 (P < 0.05) (Fig. 3B–E). Notably, no significant difference in cytokine secretion between the siRNA-β-catenin and siRNA-LKB1 intervention groups (P > 0.05) (Fig. 3B–E).

Flow cytometry revealed that the percentage of CD86-positive cells increased significantly from 8.94 ± 0.22% to 62.00 ± 1.56% after LPS intervention, whereas rhWnt1 restored the percentage of CD86-positive cells to 23.46 ± 1.35% (P < 0.05) (Fig. 3F, G). When siRNA-β-catenin, LPS and rhWnt1 were used simultaneously to treat BV2 cells, the percentage of CD86-positive cells was between that of the LPS and LPS + rhWnt1 groups (P < 0.05) (Fig. 3F, G). In contrast, the percentage of CD206-positive cells decreased significantly from 5.51 ± 0.70% to 3.25 ± 0.13% after LPS intervention, whereas rhWnt1 increased the percentage of CD206-positive cells to 23.93 ± 1.64% (P < 0.05) (Fig. 3F–H). Simultaneous treatment of BV2 cells with siRNA-β-catenin, LPS and rhWnt1 resulted in the percentage of CD206-positive cells being between that of the LPS and LPS + rhWnt1 groups (P < 0.05) (Fig. 3F–H). Similar results to siRNA-β-catenin were observed for siRNA-LKB1 in flow cytometry (P < 0.05) (Fig. 3F–H). Notably, there were no significant differences in the percentages of CD86-positive and CD206-positive cells between the siRNA-β-catenin and siRNA-LKB1 intervention groups (P > 0.05) (Fig. 3F–H).

Immunofluorescence indicated that the changes in the fluorescent intensity of CD86 and CD206 in all groups were consistent with the flow cytometry findings (Fig. 3I–K).

### Targeted silencing of β-catenin or LKB1 resulted in changes in β-catenin and NFκB-p65, LKB1, and AMPK proteins and their phosphorylation levels

**Fig. 4:**
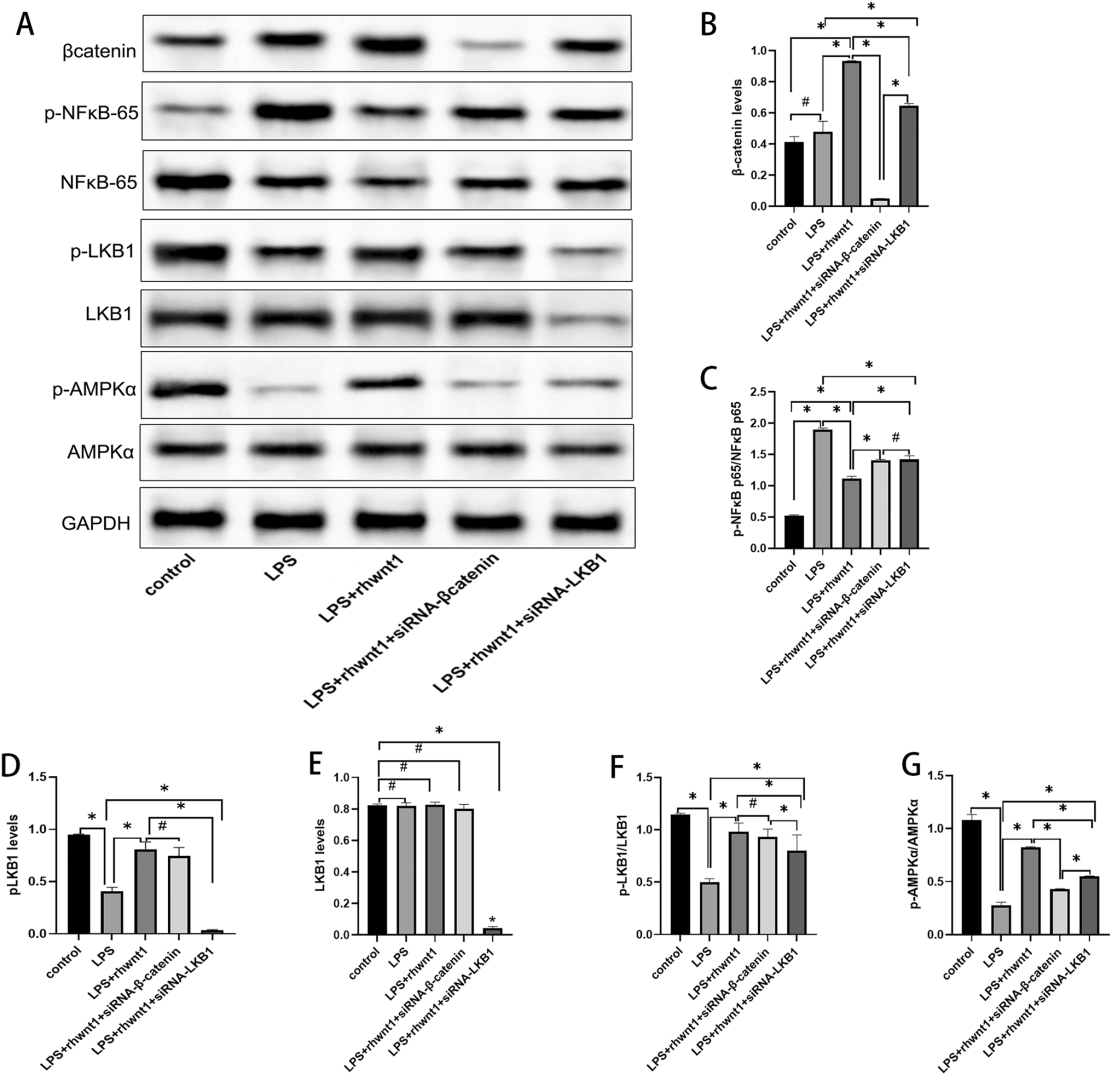
Western blotting analysis showing the changes of signaling molecules downstream of rhWnt1 after the targeted silencing of β-catenin or LKB1. The expression of β-catenin was not altered by LPS alone but was upregulated significantly by rhWnt1 in LPS-treated BV2 cells. This upregulation was inhibited by siRNA-LKB1 (**A**, **B**). rhWnt1 suppressed LPS-induced phosphorylation of NFκB-p65 in BV2 cells, which was attenuated by siRNA-β-catenin and siRNA-LKB1 (**A**, **C**). Additionally, no significant difference in NFκB-p65 phosphorylation levels was observed between the LPS + rhWnt1 + siRNA-β-catenin and LPS + rhWnt1 + LKB1 groups. β-catenin silencing did not show a significant effect on rhWnt1-induced LKB1 phosphorylation (**A**, **D**, **F**). Targeted silencing of LKB1 and β-catenin both decreased rhWnt1-induced AMPK phosphorylation to various degrees (**A**, **G**). *P < 0.05, #P > 0.05. LPS, lipopolysaccharide

Western blotting was used to evaluate the expression and phosphorylation levels of NFκB-p65, LKB1, and AMPK proteins. Although LPS alone did not alter β-catenin protein levels, BV2 cells exposed to both LPS and rhWnt1 demonstrated a 2.26 ± 0.17-fold increase in β-catenin protein levels, compared with the untreated control cells (P < 0.05) (Fig. 4A, B). In BV2 cells treated simultaneously with LPS, rhWnt1 and siRNA-β-catenin, β-catenin protein levels decreased to 0.12 ± 0.01-fold compared with those in the control group (P < 0.05) (Fig. 4A, B). When BV2 cells were co-treated with siRNA-LKB1, LPS, and rhWnt1, β-catenin expression increased 1.57 ± 0.12-fold and 0.69 ± 0.01-fold, compared with the control and LPS combined with rhWnt1 intervention groups, respectively (P < 0.05) (Fig. 4A, B).

The level of NFκB-p65 phosphorylation was increased, and the levels of LKB1 and AMPK phosphorylation decreased following LPS-induced inflammation of BV2 cells, which was attenuated by rhWnt1. Targeted silencing of β-catenin or LKB1 restricted the inhibitory effects of rhWnt1 on NFκB-p65 phosphorylation (P < 0.05) (Fig. 4A, C). Regardless of the targeted silencing of β-catenin, rhWnt1 promoted LKB1 phosphorylation that was decreased in BV2 cells treated with LPS (P < 0.05) (Fig. 4A, D, F). siRNA-LKB1 significantly reduced the level of LKB1 protein, LPS, rhWnt1 and siRNA-β-catenin had no significant effect on the level of LKB1 protein (Fig. 4E). No significant difference in LKB1 phosphorylation levels was observed between the LPS + rhWnt1 and LPS + rhWnt1 + siRNA-β-catenin groups (P > 0.05) (Fig. 4A, D, F). However, targeted silencing of LKB1 decreased the rhWnt1-induced increase in β-catenin expression as mentioned above. Targeted silencing of LKB1 or β-catenin decreased the rhWnt1-induced increase in AMPK phosphorylation to varying degrees (P < 0.05) (Fig. 4A, G).

### Effects of rhWnt1 and its downstream signals on autophagy in BV2 cells

**Fig. 5:**
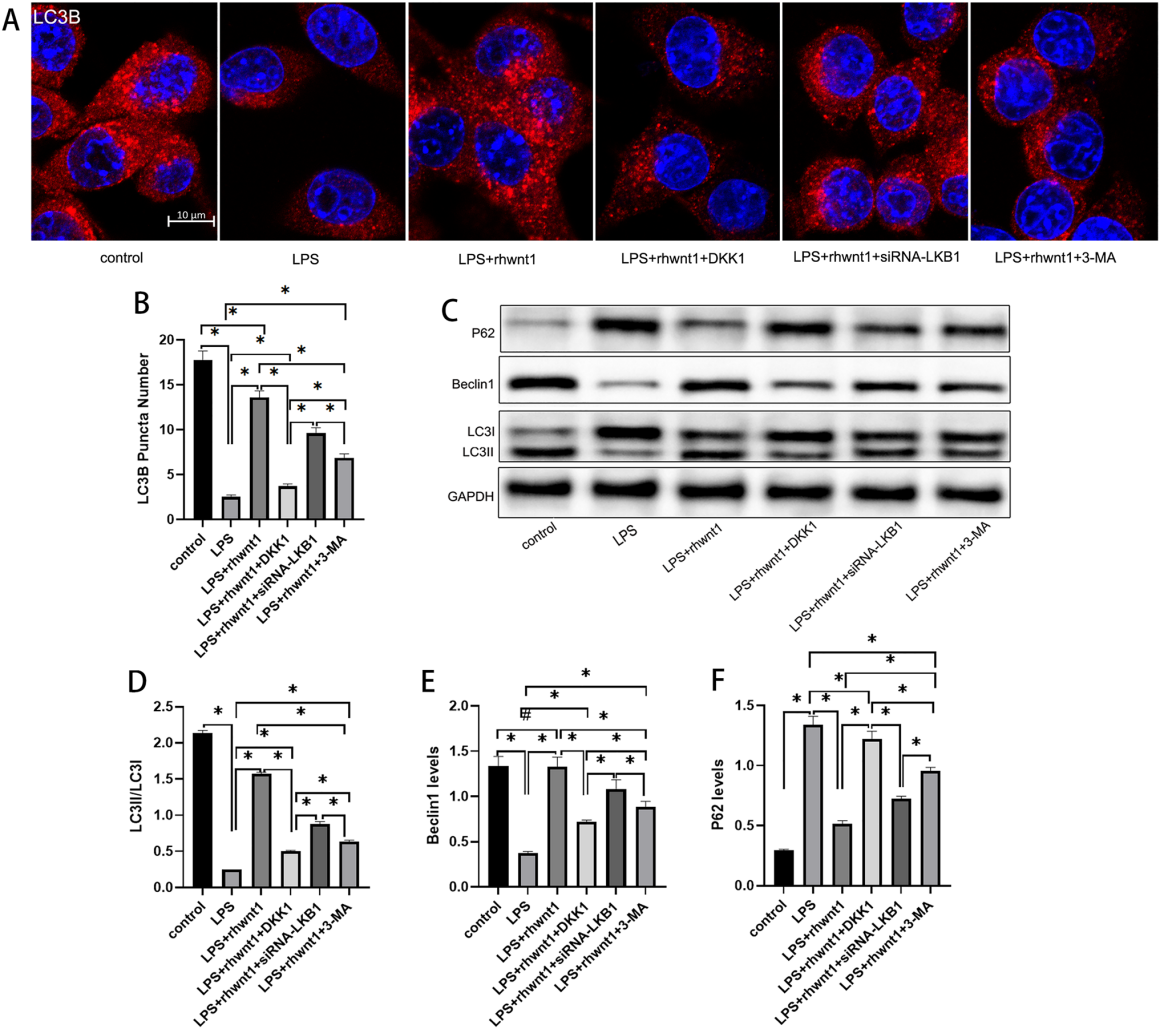
To evaluate the effects of rhWnt1 and its downstream signals on autophagy of BV2 cells, the amount of LC3B was determined by immunofluorescence assays (**A**, **B**), and the expression of P62, beclin1, and LC3 was determined by Western blotting (**C**–**F**). LPS significantly reduced the LC3B puncta number in BV2 cells, which was mitigated by rhWnt1 (**A**, **B**). DKK1 had the strongest inhibitory effect on the regulation of LC3B puncta number by rhWnt1, followed by 3-MA and siRNA-LKB1 (**A**, **B**). LPS also significantly reduced the expression ratio of LC3II/LC3I in BV2 cells, which was mitigated by rhWnt1 (**C**, **D**). DKK1 had the strongest inhibitory effect on the regulation of the expression ratio of LC3II/LC3 by rhWnt1, followed by 3-MA and siRNA-LKB1 (**C**, **D**). Although there was no significant difference in beclin1 expression between the LPS + rhWnt1 and control groups, rhWnt1 restored the decreased beclin1 expression induced by LPS (**C**, **E**). The upregulation by rhWnt1 was restrained by DKK1. The opposite trend was observed in P62 levels (**C**, **F**). *P < 0.05, #P > 0.05 LPS, lipopolysaccharide

To evaluate the effects of rhWnt1 and its downstream signals on autophagy in BV2 cells, immunofluorescence analysis of LC3B puncta number and Western blotting analysis of P62, beclin1, and LC3 were performed. Cells exposed to LPS exhibited an 86% reduction in LC3B puncta number (P < 0.05) (Fig. 5A, B). Remarkably, rhWnt1 mitigated this LPS-induced decline, restoring LC3B puncta levels to 77% of those seen in the control group (P < 0.05) (Fig. 5A, B). When BV2 cells were exposed to a combination of LPS, rhWnt1 and DKK1, the count of LC3B puncta number was merely 1.46 times greater compared with that in cells treated with LPS alone (P < 0.05) (Fig. 5A, B). Additionally, 3-MA and the targeted silencing of LKB1 only partially inhibited the LC3B-promoting effects of rhWnt1, and the inhibitory effect was smaller than that of DKK1 (P < 0.05) (Fig. 5A, B).

Compared with the control group, LPS was found to suppress the expression ratio of LC3II/LC3I in BV2 cells (P < 0.05) (Fig. 5C, D). Conversely, a notable increase in the LC3II/LC3I expression ratio was evident when BV2 cells were concurrently treated with LPS and rhWnt1, which was markedly inhibited by DKK1 (P < 0.05) (Fig. 5C, D). Meanwhile, the targeted silencing of LKB1 and 3-MA also showed a significant inhibitory effect on the elevation of the expression ratio of LC3II/LC3I induced by rhWnt1 (P < 0.05) (Fig. 5C, D). Similar results were observed in another autophagy-related molecule, beclin1, except that there was no significant difference in beclin1 expression between the LPS + rhWnt1 and control groups (Fig. 5C, E). Furthermore, the exact opposite trend was observed in P62 levels (P < 0.05) (Fig. 5C, F).

**Fig. 6:**
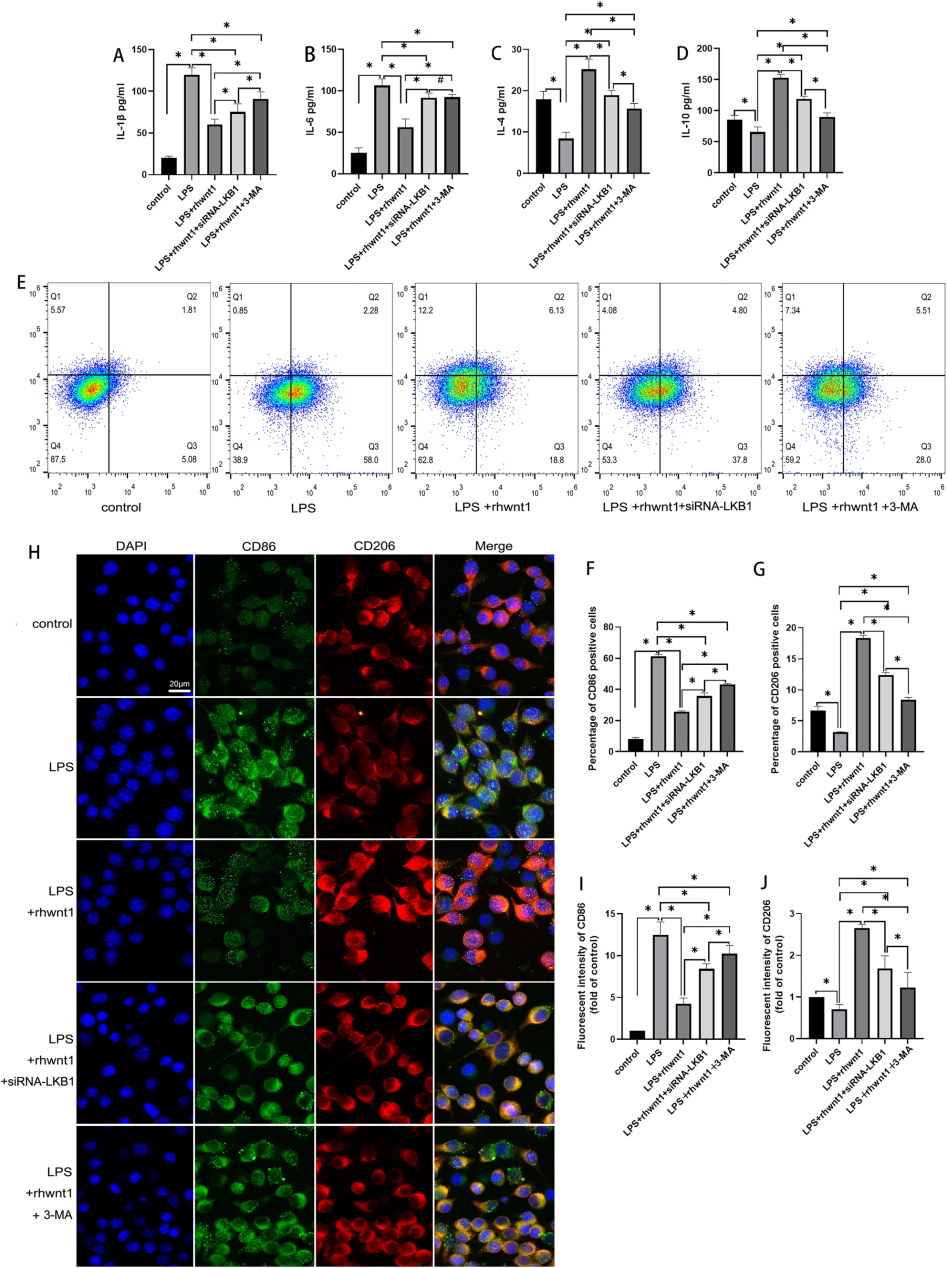
Pro- and anti-inflammatory factors were detected by ELISA. siRNA-LKB1 and 3-MA partially restricted rhWnt1 from inhibiting IL-1β and IL-6 secretion and promoting IL-4 and IL-10 secretion (**A**–**D**). The percentages of CD86-positive and CD206-positive cells were evaluated by flow cytometry and immunofluorescence. The rhWnt1-induced reduction in CD86-positive cells and increase in CD206-positive cells were partially suppressed by siRNA-LKB1 and 3-MA (**E**–**J**). *P < 0.05, #P > 0.05 LPS, lipopolysaccharide

To further determine whether Wnt1-induced promotion of BV2 cells with an M2-like phenotype was dependent on autophagy, 3-MA, an autophagy inhibitor was used to intervene in the inflammatory activation of BV2 cells. As shown in Fig. 6, siRNA-LKB1 and 3-MA incompletely restricted rhWnt1 from inhibiting IL-1β and IL-6 secretion and promoting IL-4 and IL-10 secretion (P < 0.05) (Fig. 6A–D). Flow cytometry and immunofluorescence staining revealed that the reduction in CD86-positive cells and the increase in CD206-positive cells, induced by rhWnt1, were not fully blocked by siRNA-LKB1 or 3-MA, respectively (P < 0.05) (Fig. 6E–J).

### Activated LKB1 phosphorylated AMPK

**Fig. 7:**
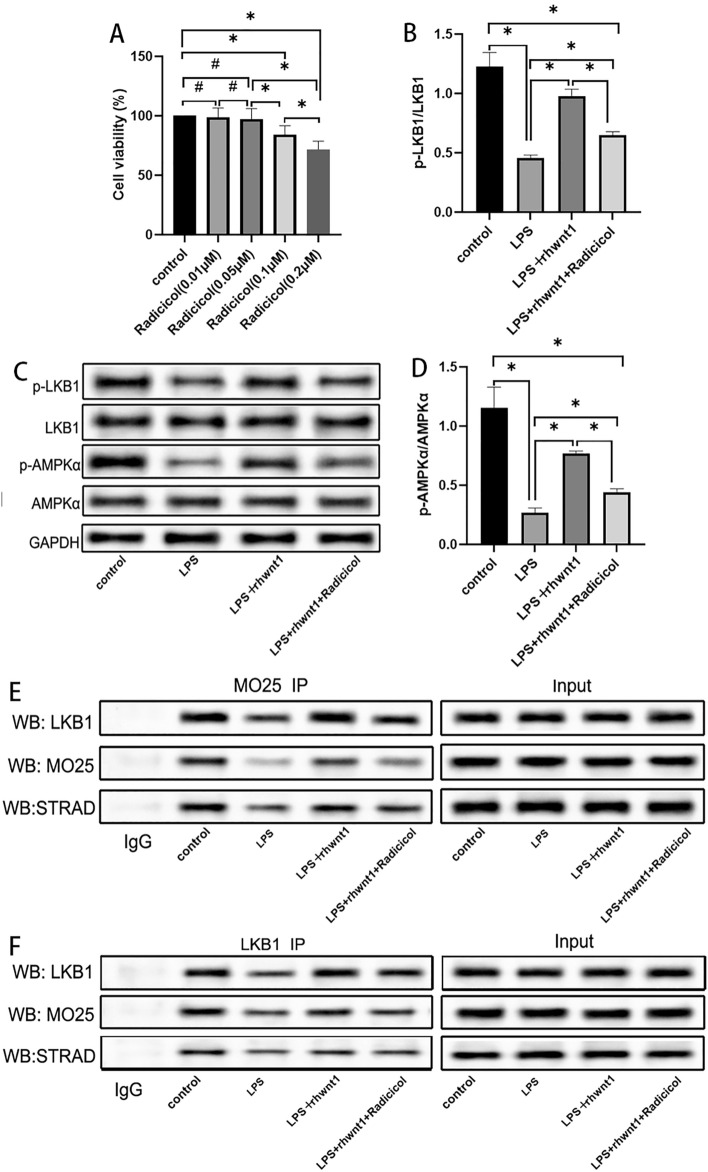
The CCK-8 assay showed 0.1 and 0.2 μM radicicol, an inhibitor of LKB1, significantly reduced the viability of BV2 cells (**A**). Western blotting demonstrated that 0.05 μM radicicol significantly inhibited the rhWnt1-induced phosphorylation of LKB1 and AMPK in LPS-treated BV2 cells (**B**–**D**). Co-immunoprecipitation showed that LPS decreased the binding capacity of LKB1, STARD, and MO25 in BV2 cells without affecting their protein expression levels, respectively. In contrast, rhWnt1 promoted the formation of the LKB1-STRAD-MO25 trimer complex, which then positively promoted its own activation (**E**, **F**). *P < 0.05, #P > 0.05 LPS, lipopolysaccharide

LKB1 binds to two proteins, STRAD and MO25, to form a trimer complex that promotes its own activation. Immunoprecipitation was used to further clarify the effects of rhWnt1 on LKB1 activation. The appropriate concentration of radicicol was first tested using the CCK-8 assay. The viability of BV2 cells was notably reduced when exposed to radicicol concentrations of 0.1 μM and 0.2 μM (P < 0.05) (Fig. 7A). Thus, BV2 cells were treated with 0.05 μM radicicol, which significantly inhibited the phosphorylation of LKB1 and AMPK induced by rhWnt1 (P < 0.05) (Fig. 7B–D). These results suggest that LKB1 activation is essential for rhWnt1 to induce AMPK phosphorylation. Although neither LPS nor rhWnt1 affected protein expression of LKB1, STARD, or MO25, the formation of the LKB1-STRAD-MO25 trimer complex was hindered by LPS, whereas rhWnt1 facilitated its development (P < 0.05) (Fig. 7E, F).

## Discussion

Numerous studies of brain damage have revealed that the volume of neuronal myelination damage is caused by disruption of oligodendrocyte proliferation and differentiation in the white matter and is accompanied by significant activation of microglial inflammation [18]. Microglial activation is influenced by Wnt factors, which can lead to varying pro-inflammatory and anti-inflammatory responses [19, 20]. In the development of the nervous system and the preservation of neuronal balance, Wnt1 is of fundamental importance [21]. Wnt1 promotes the proliferation and differentiation of neurons and oligodendrocytes and neuronal myelination [22]. In contrast, Wnt1 inhibits astrocyte and microglial activation [23], elevated Wnt1 also decreases pro-inflammatory markers to mitigate liver injury [24]. Our study revealed that the introduction of external Wnt1 protein elevated β-catenin levels and facilitated the transition of BV2 cells from a pro-inflammatory state induced by LPS to an anti-inflammatory state.

Three distinct pathways encompass the signal transduction process initiated by the attachment of the Wnt protein to a cell membrane receptor, leading to internal cellular responses: the canonical Wnt/β-catenin pathway, non-canonical planar cell polarity pathway, and Wnt/Ca2+ pathway. The same Wnt protein can activate different signal pathways in different cell types and receptors and may regulate the inflammatory response in immune cells [25]. The Wnt/β-catenin signaling cascade represents a canonical example of a signal transduction pathway. Upon binding to Frizzled and LRP5/6 receptors located on the cellular membrane, Wnt triggers a series of intracellular events. This starts with the phosphorylation of the receptor segments inside the cell. As a result, the association of downstream elements is disrupted, specifically glycogen synthase kinase-3β, axin, and phosphorylated β-catenin, leading to their dissociation. Once β-catenin separates from its complex, it undergoes dephosphorylation and moves from the cytoplasm into the nucleus, where it influences the expression of specific genes. The Wnt/β-catenin signaling pathway influences the destiny of various brain cells, such as oligodendrocyte precursor cells, microglia, astrocytes, and neurons, in a range of neuroinflammatory diseases through the action of Wnt ligands [20, 26–28]. In our study, rhWnt1 inhibited LPS-promoted inflammatory activation of microglia, and the inhibition by rhWnt1 was alleviated by DKK1. DKK1 is an LRP5/6 receptor antagonist, which prevents Wnt1 from binding to LRP5/6 receptors and disrupts the canonical Wnt signaling pathway. We speculate that Wnt1 regulates microglial inflammatory activation through the canonical Wnt signaling pathway.

The activation of microglia inflammation due to LPS relies on the phosphorylation of NFκB-p65. This process diminishes as the microglia transition from a pro-inflammatory to an anti-inflammatory state [29, 30]. In the present study, LPS-induced phosphorylation of NFκB-p65 is consistent with that reported in previous studies. Inflammatory activation of microglia was suppressed by rhWnt1, accompanied by a decrease in the phosphorylation of NFκB-p65, which was greatly inhibited by DKK1. This result, in contrast, confirmed our conjecture that Wnt1 regulates microglial inflammatory activation through the canonical signaling pathway. Furthermore, we found that rhWnt1 boosts β-catenin levels in LPS-treated BV2 cells, which were decreased after blocking with DKK1. Targeted silencing of β-catenin also mitigated the anti-inflammatory effects of Wnt1 on LPS-treated BV2 cells. Our results again show that the Wnt1/β-catenin signaling pathway is a key pathway for the anti-inflammatory effects of Wnt1.

**Fig. 8:**
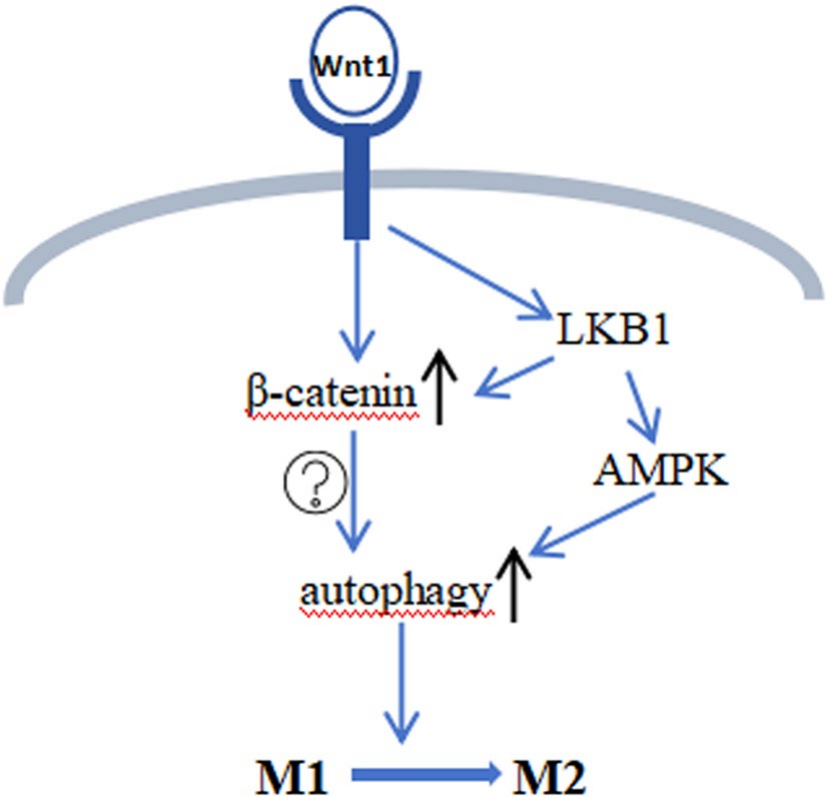
The schematic diagram showing the downstream signal transduction pathway of Wnt1 after binding to the LRP5/6 receptor. Binding of Wnt1 to the LRP5/6 receptor results in phosphorylation of LKB1 and increased β-catenin levels in the nucleus. Phosphorylated LKB1 upregulates β-catenin expression and promotes AMPK phosphorylation, thus promoting autophagy. Therefore, Wnt1 regulates microglial polarization toward the M2 phenotype by promoting autophagy

Growing evidence suggests that phosphorylation of LKB1 and AMPK promotes microglial M2 polarization [31, 32]. LKB1 is the upstream kinase that activates AMPK, and LKB1-specific knockdown inhibits AMPK phosphorylation and M1-to-M2 transformation [33]. Recent studies have shown that the reduction of inflammation by Wnt1 is linked to increased phosphorylation of LKB1 and AMPK, which was mitigated by DKK1 and radicicol. Radicicol interfered with the formation of trimers of LKB1, STARD, and MO25, and then alleviated the phosphorylation of LKB1 and AMPK. This suggests that the Wnt1/LKB1/AMPK signaling pathway is involved in the regulation of the anti-inflammatory activity by Wnt1 (Fig. 8). Furthermore, β-catenin silencing did not affect the Wnt1-induced phosphorylation of LKB1, whereas silencing of LKB1 decreased Wnt1-induced β-catenin expression. Our hypothesis suggested that the anti-inflammatory effects of the Wnt1/β-catenin pathway were influenced by LKB1. Conversely, the anti-inflammatory impacts resulting from the Wnt1/LKB1 pathway did not rely on β-catenin (Fig. 8). Previous studies have also determined that activating LKB1 can maintain stable levels of β-catenin [34].

Autophagy, as a crucial cellular catabolic pathway, is involved in maintaining various cellular homeostasis and cell survival. Emerging evidence has shown that autophagy is not only significantly suppressed in LPS-treated microglia but also acts as a negative feedback mechanism to modulate the inflammatory activity of microglia [35, 36]. Protein kinases have been shown to be essential in autophagy; thus, the regulatory role of kinases in autophagy has been investigated in several studies. The initiation of autophagy is significantly influenced by AMPK. Similarly, LKB1, the kinase that acts upstream of AMPK in the pathway, is involved in the regulation of autophagy [37]. Furthermore, modulation of microglial inflammation is also involved in autophagy activation through LKB1/AMPK signaling [38, 39]. In LPS-induced M1-like BV2 cells, the reduction in LC3B puncta number and LC3-II/LC3-I ration, alongside reduced beclin1 and elevated P62 levels, indicated the suppression of autophagy. Using the shift of LC3-I to its lipidated form, LC3-II, as a traditional marker is indicative of autophagy activity. Here, this study revealed that as the autophagic process was promoted, LPS-induced M1-like BV2 cells were inverted to an M2-like phenotype by rhWnt1, and the silencing of LKB1 and DKK1 weakened the autophagy-promoting effect of Wnt1. To further confirm that Wnt1 promoted the M2-like phenotype through the autophagy pathway, 3-MA was used to block the enhancement of autophagy induced by Wnt1, which also weakened the BV2 cell M2-like phenotype promoted by Wnt1. Therefore, it could be inferred that Wnt1 promotes the M2-like phenotype of BV2 cells by improving autophagy, and this autophagy is dependent on the LRP5/6 receptor and LKB1 pathway (Fig. 8).

We provide evidence that Wnt1 may serve as a therapeutic agent for WMI owing to its anti-inflammatory properties. However, our study has some limitations. Intrauterine infection is an important factor leading to premature birth and these premature neonates tend to experience more severe WMI, compared with neonates without infection [16, 40]. LPS has been widely used to study CNS inflammatory injury and to establish intrauterine infection models. Thus, we used LPS-treated BV2 cells to mimic the inflammatory state of WMI in preterm infants in vitro. BV2 cells are immortalized microglia and cannot represent the characteristics of preterm microglia well. To better explore the response of microglia in preterm infants stimulated by inflammation, LPS should be injected intraperitoneally into rats at 18 days of gestation. Brain tissue samples from these intrauterine infection-related premature rat pups will be used in our future studies. Additionally, the anti-inflammatory mechanism of Wnt1 should be studied in vitro using primary white matter microglia obtained from established animal models, and also in vivo using established animal models. We will focus on exploring the mechanism by which Wnt1 regulates microglia activation and its protective relevance to WMI.

Wnt1 binds to LRP5/6 receptors to regulate microglial inflammatory activation through the downstream LKB1/AMPK signaling pathway, which is independent of β-catenin and even regulates β-catenin expression. How the binding of Wnt1 and LRP5/6 receptors phosphorylates LKB1 will be the focus of further research. Following Wnt1 binding to LRP5/6 receptors, a large amount of downstream β-catenin is released into the nucleus. How β-catenin regulates autophagy and inflammatory activation is another aspect that warrants further study.

## Conclusions

Our results demonstrated that rhWnt1 suppressed microglial activation mediated by the Wnt/LRP5/6 receptor signaling pathway during LPS-induced toxicity. Furthermore, rhWnt1 promoted an M2-like phenotype in BV2 cells by enhancing autophagy, which was partly dependent on the β-catenin pathway and LKB1 phosphorylation. Although the anti-inflammatory effect of the Wnt1/LKB1/AMPK pathway was independent of β-catenin, Wnt1/LKB1 participated in the regulation of β-catenin. Our research revealed a new process by which Wnt1 reduces inflammation in BV2 cells activated by LPS.

## Supplementary Information

Below is the link to the electronic supplementary material.

Supplementary file1 (DOCX 5640 KB)

## Data Availability

Data related to the study can be provided by the corresponding author when requested appropriately.

## Abbreviations

CNS: Central nervous system
LPS: Lipopolysaccharide
WMI: White matter injury
LKB1: Liver Kinase B1
AMPK: AMP-activated protein kinase
DKK1: Dickkopf related protein 1
