# Supplementary material for: Wnt1 oversees microglial activation by the Wnt/LRP5/6 receptor signaling pathway during lipopolysaccharide-mediated toxicity
Source: Mol Biol Rep. 2025 Mar 1;52(1):273. doi: 10.1007/s11033-025-10360-2 (PMC11872766; doi:10.1007/s11033-025-10360-2)
Supplement: Supplementary file 1 — Supplementary file1 (DOCX 5640 KB) [file 11033_2025_10360_MOESM1_ESM.docx]

**
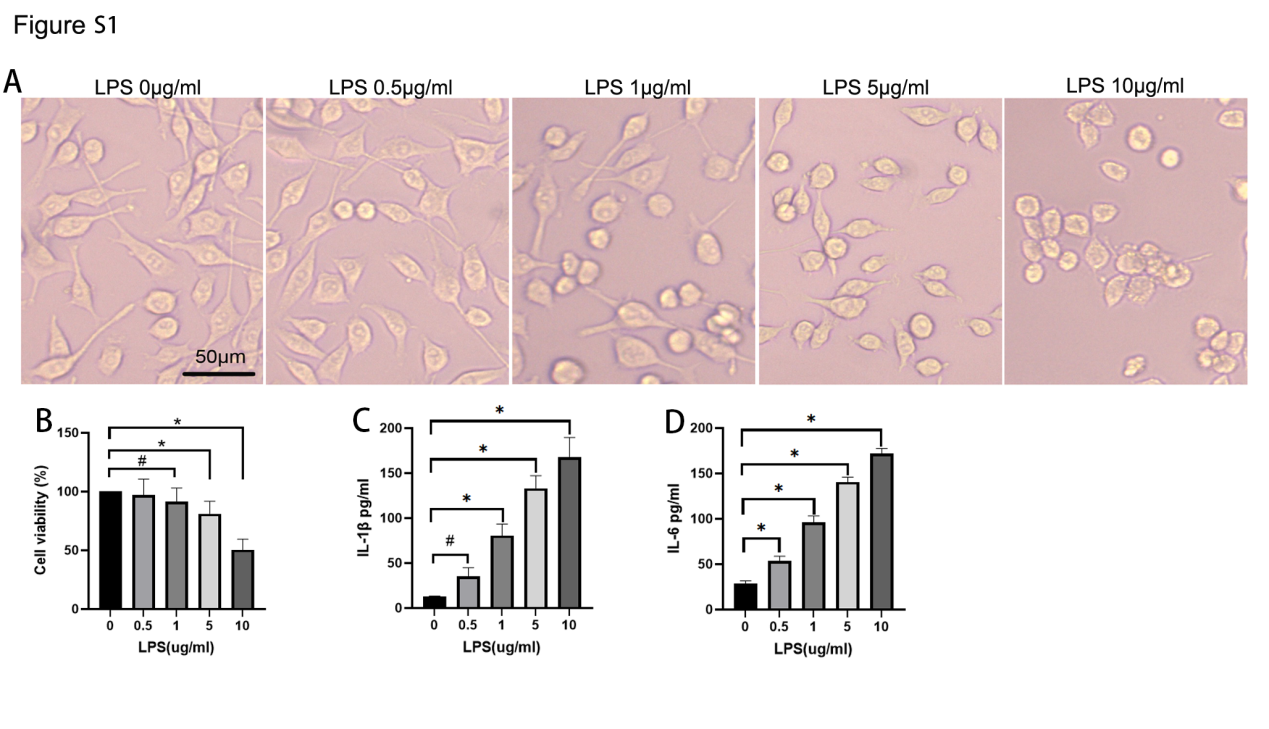
**

**Fig. S1** LPS promoted inflammatory activation of BV2 cells. Morphology of BV2 cells in response to different concentrations of LPS (0, 0.5, 1, 5, 10 μg/mL) for 2 h (A). Following incubation with 0.5 and 1 μg/mL LPS, BV2 cells showed enlarged cell body, enlarged proximal antenna, and reduced distal antenna branching. When incubated with 5 and 10 μg/mL LPS, BV2 cells showed shrunken bodies and reduced branching. Cell viability was detected by the CCK-8 assay. A significant reduction in cell viability was observed when the LPS concentration reached 5 μg/mL, and the higher the concentration, the worse the viability of BV2 cells (B). The secretion of pro-inflammatory cytokines (IL-1β, IL-6) was detected by ELISA. Although 0.5 µg/mL LPS did not alter the level of IL-1β, the higher the concentration, the more proinflammatory cytokines were secreted (C, D). Error bars represent SEM. *P<0.05, #P>0.05. LPS, lipopolysaccharide; ELISA, enzyme-linked immunosorbent assay

**
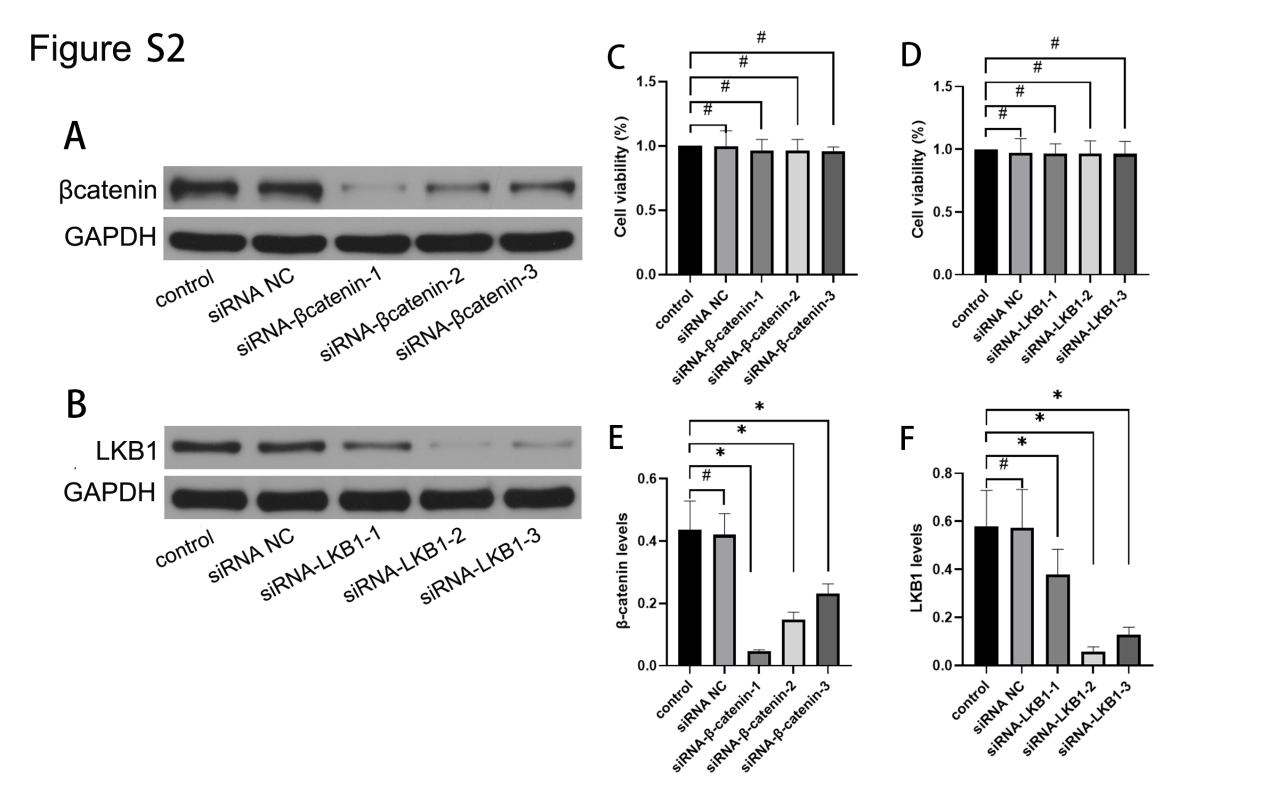
**

**Fig. S2** Silencing efficiencies of siRNA-β-catenin and siRNA-LKB1 were detected by western blotting. The silencing efficiencies of siRNA-β-catenin-1 and siRNA-LKB1-2 were the most significant in the different siRNA intervention groups (A, B, E, F). Cell viability was detected by the CCK-8 assay. No significant differences in cell viability were detected across the groups (C, D). *P<0.05, #P>0.05
